# Genome-Wide Association Study Identifying Candidate Genes Influencing Important Agronomic Traits of Flax (*Linum usitatissimum* L.) Using SLAF-seq

**DOI:** 10.3389/fpls.2017.02232

**Published:** 2018-01-09

**Authors:** Dongwei Xie, Zhigang Dai, Zemao Yang, Jian Sun, Debao Zhao, Xue Yang, Liguo Zhang, Qing Tang, Jianguang Su

**Affiliations:** ^1^Institute of Bast Fiber Crops, Chinese Academy of Agricultural Sciences, Changsha, China; ^2^Institute of Industrial Crops, Heilongjiang Academy of Agricultural Sciences, Harbin, China; ^3^College of Agriculture, Northeast Agricultural University, Harbin, China

**Keywords:** flax (*Linum usitatissimum* L.), agronomic traits, GWAS, SLAF-seq, candidate genes

## Abstract

Flax (*Linum usitatissimum* L.) is an important cash crop, and its agronomic traits directly affect yield and quality. Molecular studies on flax remain inadequate because relatively few flax genes have been associated with agronomic traits or have been identified as having potential applications. To identify markers and candidate genes that can potentially be used for genetic improvement of crucial agronomic traits, we examined 224 specimens of core flax germplasm; specifically, phenotypic data for key traits, including plant height, technical length, number of branches, number of fruits, and 1000-grain weight were investigated under three environmental conditions before specific-locus amplified fragment sequencing (SLAF-seq) was employed to perform a genome-wide association study (GWAS) for these five agronomic traits. Subsequently, the results were used to screen single nucleotide polymorphism (SNP) loci and candidate genes that exhibited a significant correlation with the important agronomic traits. Our analyses identified a total of 42 SNP loci that showed significant correlations with the five important agronomic flax traits. Next, candidate genes were screened in the 10 kb zone of each of the 42 SNP loci. These SNP loci were then analyzed by a more stringent screening via co-identification using both a general linear model (GLM) and a mixed linear model (MLM) as well as co-occurrences in at least two of the three environments, whereby 15 final candidate genes were obtained. Based on these results, we determined that *UGT* and *PL* are candidate genes for plant height, *GRAS* and *XTH* are candidate genes for the number of branches, *Contig1437* and *LU0019C12* are candidate genes for the number of fruits, and *PHO1* is a candidate gene for the 1000-seed weight. We propose that the identified SNP loci and corresponding candidate genes might serve as a biological basis for improving crucial agronomic flax traits.

## Introduction

Flax (*Linum usitatissimum* L.) is one of the oldest plants cultivated for fiber and edible oil and remains an important cash crop worldwide. Breeding selection for fiber flax or linseed flax has resulted in two plant types, which differ considerably in agronomic performance (Diederichsen and Ulrich, 2009). Compared with linseed flax cultivars, fiber flax plants are typically taller, with fewer branches and fruits and lower seed production (Booth et al., 2004). Therefore, agronomic traits directly affect the seed yield of linseed flax and the bast fiber quality of fiber flax. In recent years, traditional breeding methods have been employed to introduce genetic changes that improve the agronomic traits of flax. However, agronomic features are complex, quantitative traits that are controlled by multiple genes. Consequently, traditional breeding approaches do not satisfy the demand for improving flax traits. Thus, far a number of genetic studies on agronomic traits of flax have been reported. For example, amplified fragment length polymorphism (AFLP) and simple-sequence repeat (SSR) markers were used to perform QTL analysis for four flax traits, revealing several yield-related QTLs (Gehringer et al., 2006). In addition, 464 SSR markers were employed to perform QTL analysis for nine traits in a natural population composed of 390 flax germplasm resources that were planted in eight environments (Soto-Cerda et al., 2014); in that study, the authors identified 12 markers that were closely linked to six traits. A genome-wide scan was performed using 407 core germplasm resources and 448 SSR markers before association mapping was conducted to elucidate the non-neutral genomic regions potentially underlying divergent selection between fiber and linseed cultivars, and the candidate genes involved in the biosynthesis of the cell wall, lignin, and fatty acids were analyzed (Soto-Cerda et al., 2013). Furthermore, Deng et al. (2014) used 61 pairs of SSR primers, 91 pairs of expressed sequence tag (EST)-SSR primers, and 102 pairs of genomic-SSR primers to perform association analysis for yield-related traits in 182 core germplasm resources of flax; the authors identified 57 high-quality allelic variations, including 31 showing yield-enhancing effects and 26 showing the opposite. Nevertheless, these association studies were all based on common molecular markers (e.g., SSR, EST-SSR) that might not be sufficient in light of the rapid development of new sequencing technologies. Furthermore, the investigations cited above were not enough to elucidate the genes related to the complicated agronomic traits of flax.

In recent years, genome-wide association studies (GWAS) based on next-generation sequencing (NGS) technology have become the new approach for improving crop traits. GWAS is suitable for phenotypic data under multiple environments, thereby reducing environment-induced errors and enhancing results accuracy (Hall et al., 2010). Once the genotype data of a population are available, GWAS can be performed to examine multiple traits (Atwell et al., 2010). As such, the approach provides a basis for elucidating the genetic structures of the complex traits of a crop. The resulting association alleles can be used for marker-assisted molecular breeding and are crucial for innovating germplasm resources and improving cultivars. So far, GWAS related to important agronomic traits have been reported in several crops, including maize (Xue et al., 2013; Farfan et al., 2015), rice (Huang et al., 2010; Han et al., 2016), and soybean (Sonah et al., 2015; Zhang et al., 2015). However, an NGS-based GWAS analysis examining agronomic traits in flax (*L. usitatissimum*) has not been reported.

Previous GWAS have mostly been based on SNP array technology, which can detect known SNP loci but not new loci (Vilkki et al., 2013; Zhang et al., 2016). In light of this limitation, a high-throughput sequencing-based technology known as specific locus-amplified fragment sequencing (SLAF-seq) was developed (Sun et al., 2013). In comparison with other technologies, SLAF-seq has the following advantages: (i) generation of high-density SNP loci numbering in the millions after one sequencing reaction, (ii) capability of detecting novel SNP loci in unknown mutation-harboring loci compared with SNP arrays, (iii) suitability for any species regardless of the presence of a reference genome, and (iv) a higher rate of identified SNP loci that become genuine association markers. As a consequence, the technology has been applied to many crops including rice, soybean, sesame, cucumber, *Brassica napus*, etc. (Zhang et al., 2013; Xu et al., 2014; Geng et al., 2016; Han et al., 2016; Li et al., 2016).

Here, we examined 224 core germplasm resources of flax grown under different environmental conditions for the main agronomic traits of plant height, technical length, number of branches, number of fruits, and 1000-grain weight. Subsequently, SLAF-seq was employed to perform GWAS and recover potential alleles controlling these traits. To our knowledge, this is the first SLAF-seq-based GWAS with the goal to identify SNP loci and candidate genes linked to important agronomic flax traits. The results provide a basis for molecular marker (related to main agronomic traits)-assisted breeding and improvement of the main agronomic traits in flax.

## Materials and methods

### Experimental materials and survey of traits

The core germplasm resources of 224 flax accessions were collected from institutions in China and other countries (Table S1). They were sown at the Harbin Experimental Base of Heilongjiang Academy of Agricultural Sciences (45°65′N, 126°68′E), Harbin, China, in April of 2015 and 2016 (2015HRB, 2016HRB) as well as at the Lanxi Experimental Base (6°27′N, 126°28′E), Lanxi, China, in April of 2016 (2016LX). The average annual rainfall from seeding to harvest was 390.1 mm in 2015HRB and 453 mm in 2016HRB, and the average annual temperature was 5.26° and 4.99°C. The average annual rainfall from seeding to harvest was 504.4 mm in 2016LX, and the average annual temperature was 3.11°C. The experiment at each location used a randomized completed block design with three replicates. Each cultivar was planted in triplicate in 2-m lines, with a 20-cm inter-line gap. The field management was the same as the local field management. At the maturing stage, ten plants were selected from each replicate for phenotyping. The five agronomic traits (plant height, technical length, branch number, fruit number, 1,000-grain weight) were investigated. Plant height was measured as the distance between the cotyledon scar and the top of the first-degree branch. Technical length was measured as the distance between the cotyledon scar of the flax plant and the base of the first-degree branch below the inflorescence. The branch number was the number of first-degree branches on the top of the main stem. The fruit number was the number of all fruits that had seeds on the top of the main stem. The 1,000-grain weight was the absolute weight of 1,000 seeds (water content 9%) that were mature, full and clean.

### Extraction of genomic DNA

Genomic DNA was isolated from fresh leaves harvested from 20-day-old seedlings of flax. The Tiangen plant total genomic DNA extraction kit (Tiangen Biotech Co. Ltd., Beijing, China) was used for the genomic DNA extraction. A NanoDrop 2000 (Thermo Scientific, Massachusetts) was used to determine the DNA concentration and quality to ensure that DNA samples met the requirements of the sequencing reaction (concentration ≥ 18 ng/μL; volume ≥ 30 μL).

### Modification of the genomic DNA

First, the restriction enzyme HaeIII was used to digest the genomic DNA in a 50 μL aqueous solution containing 500 ng of genomic DNA, 41 μL of NEB Buffer (10 ×), and 0.12 μL of HaeIII (1 U/μL). This solution was incubated at 37°C for 15 h. The resulting DNA was column-purified using a QIAGEN kit and solubilized in 50 μL of EB (0.01 mol/L). The sticky ends of the digested DNA fragments were filled in, and their 5′ ends were then phosphorylated using a 100 μL solution containing 30 μL of purified DNA (50 ng/μL), 10 μL of T4 DNA Ligase Buffer (containing 10 mmol/L final concentration ATP), 4 μL dNTP Mix (10 mmol/L), 5 μL of T4 DNA polymerase (5 U/μL), 1 μL of Klenow fragment (5 U/μL), and 5 μL of T4 polynucleotide kinase (10 U/μL). This solution was incubated at 20°C for 30 min in a thermocycler. The DNA was column-purified using a QIAGEN kit and solubilized in 33 μL of EB (0.01 mol/L). Next, a base was added to the 3′ ends of the 5′ phosphorylated DNA fragments, allowing them to connect to the Solexa adaptor, which has a T base at its 5′ end, using the following conditions: 32 μL of purified DNA (50 ng/μL), 5 μL of Klenow Buffer (10 ×), 10 μL of dATP (1 mmol/L), and 3 μL of Klenow Exo (5 U/μL). The solution was placed in a 37°C water bath for 30 min. The DNA was column-purified using a QIAGEN kit and solubilized in 10 μL of EB (0.01 mol/L). Finally, the Solexa adapter was attached to the DNA fragments to allow them to hybridize in the flow cells in the sequencing reactions. The reaction conditions were as follows: the solution containing 10 μL of purified DNA (50 ng/μL), 25 μL of DNA Ligase Buffer (2 ×), 10 μL of Adapter (5 pmol/μL), and 5 μL of DNA Ligase (5 U/μL) was incubated at 20°C for 15 min in the polymerase chain reaction (PCR) system. The DNA was column-purified using a QIAGEN kit and solubilized in 30 μL of EB (0.01 mol/L).

### PCR amplification and sequencing

Based on the restriction analysis of PAProC (http://www.paproc.de/) (Nussbaum et al., 2001), DNA fragments of 500–580 bp were gel purified and PCR amplified using a forward primer (5′-AATGATACGGCGACCACCGA-3′) and a reverse primer (5′-CAAGCAGAAGACGGCATACG-3′). PCR amplification was performed in a 40 μL aqueous solution containing 8 μL of purified DNA (50 ng/μL), 1.5 μL of forward primer (50 pmol/μL), 1.5 μL of reverse primer (50 pmol/μL), 9 μL of dNTP mix (10 mmol/L) and 20 μL of Phusion DNA polymerase (2 U/μL). The amplification procedure was as follows: pre-denaturation at 98°C for 30 s, followed by 18 amplification cycles of denaturation at 98°C for 10 s, annealing at 65°C for 30 s, and extension at 72°C for 30 s, before a final extension at 72°C for 5 min. After the reaction, the DNA was column-purified using a QIAGEN kit and solubilized in 30 μL of EB (0.01 mol/L). Purified DNA samples were quantified using the Qubit system before bridge amplification was performed on the surface of the flow cells to generate DNA clusters. The PCR products were re-purified and then prepared for paired-end sequencing on an Illumina HiSeq 2500 sequencing platform (Illumina, San Diego, CA, USA).

### Data processing and data submission information

Raw sequencing reads were separated using barcode sequences: Illumina SLAF libraries were barcoded with standard Illumina multiplex adaptors and pooled for sequencing in sets of three samples to generate an average of 6-fold sequence coverage per sample (Purcell et al., 2007; Healey et al., 2014). Low-quality reads (QC score < 20) were removed before SOAP 2.20 (Sun et al., 2013) was employed to align the resulting reads with the reference genome of *Linum usitatissimum* v1.0 (https://phytozome.jgi.doe.gov/pz/portal.html#!search?show=KEYWORD) (Wang et al., 2012). A read was considered valid if both ends mapped onto the genome and could be used to define SLAF markers. Based on the results of alignment and correction, groups with a mean sequencing depth of 4 were recruited to define SLAF markers. Next, the number of SLAF markers per 100 K genome was recorded to obtain the distribution of SLAF markers in the scaffolds. Finally, SNP loci were detected in the collection of the 224 specimens using pre-defined SLAF markers, whereby the number of SNP loci per 100 K genome was documented. Raw Illumina sequences were deposited in the National Center for Biotechnology Information (NCBI) and can be accessed in the database (https://www.ncbi.nlm.nih.gov/) under accession SRP116365 or SRS2474942 for leaf.

### Population structure analysis and significant SNP discovery

The population structure analysis used 146,959 SNPs to infer the genetic background of an accession that belongs to a cluster under a given number of populations (K). The number of genetic clusters was predefined as *K* = 1–5 for all accessions and was calculated using Admixture software (Hardy and Vekemans, 2002; Alexander et al., 2009).

LD (linkage disequilibrium) between pairs of SNPs was estimated by using squared allele frequency correlations (*r*^2^) in Tassel version 3.0 (Bradbury et al., 2007). Each significant SNP was evaluated for the extent of local LD. The region was defined as extending to where LD between nearby SNPs and the lead SNP decayed to *r*^2^ > 0.8, MAF > 0.05. Only SNPs with an MAF more than 0.05 and <10% missing data were used. The SNP nomenclature used in this study is based on the number of scaffolds that contained an SNP plus the position of the polymorphism in the scaffold.

### Genome-wide association analyses

The efficient model was performed with both GLM and MLM using Tassel software. The population structure matrix generated from Admixture was used as the Q matrix for the GLM model. *P*-values of *P* ≤ 1.268 × 10^−5^ (*P* = 0.01/n; *n* = total markers used, which is roughly a Bonferroni correction, corresponding to -log10 (*P*) = 5, red line) and *P* ≤ 1.268 × 10^−6^ (*P* = 0.1/n; *n* = total markers used, which is roughly a Bonferroni correction, corresponding to -log10 (*P*) = 6, blue line) were defined as the genome-wide control threshold and suggestive threshold, respectively. The genes within 10 Kb of a significant SNP's flanking region were reported as candidate genes.

## Results

### Descriptive statistics of agronomic traits

Under three different environmental conditions, plant height had a minimum value of 42.20 cm, a maximum value of 125.40 cm, and a maximum coefficient of variation of 18.09%; technical length had a minimum value of 27.60 cm, a maximum value of 103.20 cm, and a maximum coefficient of variation of 22.76%; number of branches had a minimum value of 2, a maximum value of 12, and a maximum coefficient of variation of 55.57%; number of fruits had a minimum value of 2, a maximum value of 39, and a maximum coefficient of variation of 53.47%; and 1,000-grain weight had a minimum value of 3.18 g, a maximum value of 9.21 g, and a maximum coefficient of variation of 16.27%. The results therefore indicated that the test germplasm resources of flax contained extraordinary genetic variation (Table 1).

**Table 1:** Results of five important agronomic traits derived from the flax germplasm resources.

**Environments**	**Traits**	**Minimum value**	**Maximum value**	**Range**	**Mean value**	**Standard deviation**	**Coefficient of Variation (%)**
2015HRB	Plant height (cm)	42.20	109.50	67.30	81.94	14.82	18.09
	Technical length (cm)	27.60	94.80	67.20	64.38	14.27	22.19
	Number of branches (unit)	2.00	12.00	10.00	4.50	1.08	24.00
	Number of fruits (unit)	4.00	29.00	25.00	9.00	3.73	41.44
	1,000-grain weight (g)	3.94	8.91	4.97	5.00	0.77	15.32
2016HRB	Plant height (cm)	49.50	120.10	70.60	89.77	15.65	17.43
	Technical length (cm)	35.20	103.20	68.00	70.52	16.05	22.76
	Number of branches (unit)	2.00	12.00	10.00	4.00	1.13	28.25
	Number of fruits (unit)	2.00	30.00	29.00	6.16	3.12	50.64
	1,000-grain weight (g)	3.18	8.92	5.74	4.96	0.79	15.92
2016LX	Plant height (cm)	54.20	125.40	71.2	91.45	14.46	15.81
	Technical length (cm)	30.30	100.40	70.1	65.81	14.92	22.67
	Number of branches (unit)	2.00	10.00	8.00	5.47	3.04	55.57
	Number of fruits (unit)	4.00	39.00	35.00	10.06	5.38	53.47
	1,000-grain weight (g)	3.82	9.21	5.39	5.10	0.83	16.27

### Sequencing results

SNP detection was performed in the collection of 224 germplasm resources of flax based on the predefined 346,639 SLAF tags, which generated a total of 584,987 SNP loci (MAF ≥ 0.05). Considering both the SLAF and SNP data, we defined the SLAF markers associated with SNPs as polymorphic SLAF markers to thereby examine the SLAF polymorphisms. Our analysis yielded a total of 146,959 polymorphic SLAF markers with a mean depth of 7.2. After quality control, there were 34,932 SNP loci used for subsequent GWAS analyses (Table S2).

### Analysis of population structure

**Figure 1:**
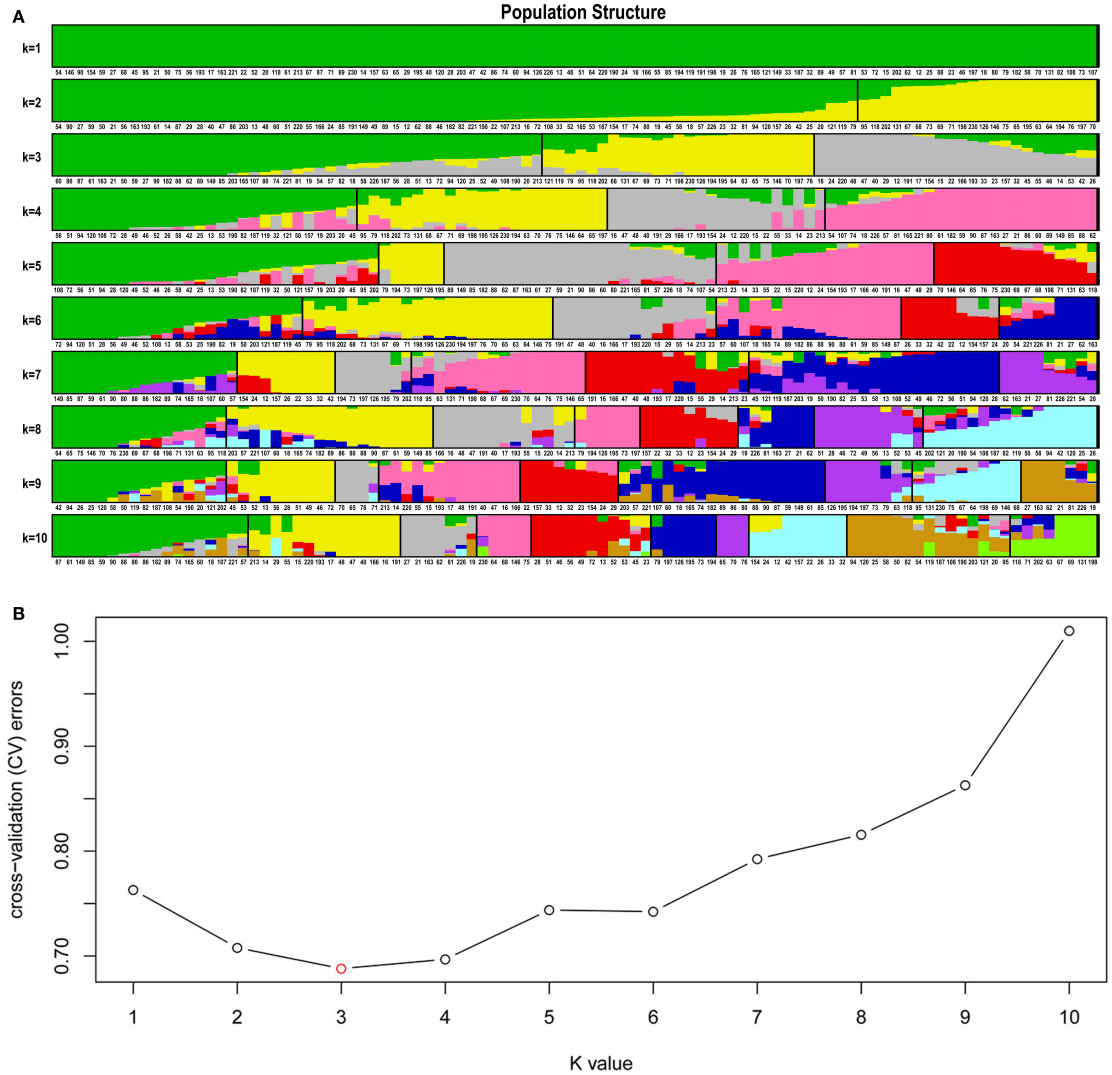
Cluster analysis and population structure of 224 germplasm resources of flax. **(A)** Cluster map of the population structure (each color represents a cluster, and each row represents the result of a given number of clusters (K, *K* = 1–10); **(B)** Population structure plot [ordinate represents cross-validation error rate (*CV*-value); abscissa represents number of clusters (K)].

The Admixture software was used to analyze the population clustering and structure of the 224 germplasm resources (Figures 1A,B). Specifically, clustering was first performed assuming that the number of clusters (K) was between 1 and 10. Then, the results were cross-validated to determine that the optimal *K*-value was 3 (according to the valley of the error rates of cross-validation). In other words, our results implied that the collection most likely originated from three ancestors. Given that population stratification might affect the accuracy of association analysis, we generated QQ plots of individual traits (Supplementary Figures 1–5). The results indicated that the observation values (ordinate) generally matched with the corresponding expected values (abscissa), suggesting that the association analysis did not produce any false negativity due to population stratification. Hence, the GWAS results were reliable.

### Genome-wide association analysis

#### SNP loci displaying significant correlation with plant height

The GLM and MLM models of TASSEL were employed to perform GWAS, which revealed that nine SNP loci were significantly associated with plant height (*P* < 1.26E-06). The relevant Manhattan plots and QQ plots of the two models and three environments are shown in Supplementary Figure 1. The GLM generated nine SNP loci in the three environments, including six in 2015HRB, one in 2016HRB, and two in 2016LX. However, there was not a single SNP that occurred in more than one environment. In comparison, the MLM only generated two SNP loci (scaffold344_309662 and scaffold51_1349321) in 2015HRB, both of which were also identified by the GLM in 2015HRB. The genes closest to the two SNP loci include *UGT* (UDP-glycosyltransferase) and *PL* (Pectate lyase). Moreover, the genes closest to the other seven SNP loci were *CBP* (Calcineurin B-like protein), *PI-PLC X* (PI-PLC X domain-containing protein), *SPP* (Squamosa promoter-binding-like protein), *PPR* (PPR repeat family), *PSP* (Pectate lyase superfamily protein), *UF* (Ubiquitin family), and *CS* (Cellulose synthase) (Table 2).

**Table 2:** Associated single nucleotide polymorphisms (SNPs) and the nearest genes for plant height traits of flax.

**Model**	**Environment**	**SNP position (bp)**	**scaffold**	**Location**	***P*-value**	**Nearest gene**	**Distance to SNP (kb)**
GLM	2015HRB	scaffold112_114241	scaffold112	114241	7.43E-07	Calcineurin B-like protein (CBP)	upstream 0.27
		scaffold1491_318496	scaffold1491	318496	1.91E-07	PI-PLC X domain-containing protein (PI-PLC X)	interior
		scaffold31_1800846	scaffold31	1800846	6.65E-07	Squamosa promoter-binding-like protein (SPP)	downstream 0.642
		scaffold344_309662	scaffold344	309662	1.11E-07	UDP-glycosyltransferase 1 (UDP)	downstream 4.558
		scaffold51_1349321	scaffold51	1349321	8.08E-07	Pectate lyase (PL)	downstream 8.566
		scaffold59_572553	scaffold59	572553	1.06E-07	PPR repeat family (PPR)	upstream 0.52
	2016HRB	scaffold156_641874	scaffold156	641874	7.27E-07	Pectate lyase superfamily protein (PSP)	downstream 0.51
	2016LX	scaffold147_367986	scaffold147	367986	1.10E-07	Ubiquitin family (UF)	upstream 8.3
		scaffold859_123972	scaffold859	123972	4.22E-07	cellulose synthase (CS)	downstream 0.56
MLM	2015HRB	scaffold344_309662	scaffold344	309662	1.11E-07	UDP-glycosyltransferase (UDP)	downstream 4.558
		scaffold51_1349321	scaffold51	1349321	8.08E-07	Pectate lyase (PL)	downstream 8.566

#### SNP loci displaying significant correlation with technical length

Three SNP loci, identified by only the GLM in two environments, were found to be significantly associated with technical length (*P* < 1.26E-06) (Supplementary Figure 2). Among these, scaffold297_275113 and scaffold361_14957 were identified in 2015 HRB, whereas scaffold273_68457 was identified in 2016 HRB. Genes closest to the three SNP loci included *HP* (Hypothetical protein), *VTP* (Vesicle transport protein), and *MIF* (Macrophage migration inhibitory factor) (Table 3).

**Table 3:** Associated single nucleotide polymorphisms (SNPs) and the nearest genes for the technical length trait of flax.

**Model**	**Environment**	**SNP position (bp)**	**scaffold**	**Location**	***P*-value**	**Nearest gene**	**Distance to SNP (kb)**
GLM	2015HRB	scaffold297_275113	scaffold297	275113	3.96E-07	hypothetical protein(HP)	downstream 1.83
		scaffold361_14957	scaffold361	14957	9.44E-07	Vesicle transport protein(VTP)	upstream 1.19
	2016HRB	scaffold273_68457	scaffold273	68457	1.18E-06	Macrophage migration inhibitory factor(MIF)	interior

#### SNP loci displaying significant correlation with number of branches

Twenty-one SNP loci exhibited a significant association with the number of branches (*P* < 1.26E-06). The relevant Manhattan plots and QQ plots of the two models and three environments are shown in Supplementary Figure 3. GLM identified nine SNP loci in 2015HRB, two SNP loci in 2016HRB, and nine SNP loci in 2016LX. Among these, eight SNP loci were identified in both 2015HRB and 2016LX (scaffold116_30201, scaffold156_1203677, scaffold1863_545, scaffold353_773806, scaffold42_494571, scaffold464_754364, scaffold635_43971, and scaffold977_784147). MLM identified six SNP loci in 2015HRB, two SNP loci in 2016HRB, and seven SNP loci in 2016LX. Among these, six SNP loci were identified in both 2015HRB and 2016LX (scaffold116_30201, scaffold156_1203677, scaffold1863_545, scaffold353_773806, scaffold464_754364, and scaffold977_784147). There were eight SNP loci co-identified by both the GLM and MLM (scaffold116_30201, scaffold156_1203677, scaffold1863_545, scaffold353_773806, scaffold464_754364, scaffold977_784147, scaffold359_282990, and scaffold359/289139). In addition, six SNP loci displayed associations in both models and were identified in two environments (2015HRB and 2016LX) (scaffold116_30201, scaffold156_1203677, scaffold1863_545, scaffold353_773806, scaffold464_754364, and scaffold977_784147). The genes closest to the six SNP loci were *GRAS* (GRAS domain family), *GST* (Glutathione S-transferase), *PORR* (Plant organelle RNA recognition domain), *PIP5K* (Phosphatidylinositol-4-phosphate 5-Kinase), *XTH* (Xyloglucan endotransglucosylase/hydrolase), and *DDR* (DNA-damage-repair) (Table 4).

**Table 4:** Associated single nucleotide polymorphisms (SNPs) and the nearest genes for the number of branches trait of flax.

**Model**	**Environment**	**SNP position (bp)**	**scaffold**	**Location**	***P*-value**	**Nearest gene**	**Distance to SNP (kb)**
GLM	201HRB	scaffold116_30201	scaffold116	30201	3.86E-11	GRAS domain family (GRAS)	upstream 9.57
		scaffold156_1203677	scaffold156	1203677	2.29E-11	Glutathione S-transferase (GST)	downstream 0.52
		scaffold1863_545	scaffold1863	545	8.39E-11	Plant organelle RNA recognition domain (PORR)	upstream 6.46
		scaffold212_601171	scaffold212	601171	1.63E-07	Cytochrome P450 (P450)	upstream 4.36
		scaffold353_773806	scaffold353	773806	7.04E-11	Phosphatidylinositol-4-phosphate 5-Kinase (PIP5K)	downstream 6.62
		scaffold42_494571	scaffold42	494571	1.79E-07	Glycerophosphodiester phosphodiesterase (GP)	interior
		scaffold464_754364	scaffold464	754364	7.77E-07	xyloglucan endotransglucosylase/hydrolase (XTH)	interior
		scaffold635_43971	scaffold635	43971	1.08E-06	Ricinus communis acid phosphatase (RCAP)	interior
		scaffold977_784147	scaffold977	784147	2.69E-10	DNA-damage-repair (DDR)	downstream 1.65
	2016HRB	scaffold212_216830	scaffold212	216830	6.81E-07	Transferase family (TF)	upstream 6.80
		scaffold359_282990	scaffold359	282990	7.47E-12	Aldehyde dehydrogenase (AD)	interior
	2016LX	scaffold116_30201	scaffold116	30201	3.86E-11	GRAS domain family (GRAS)	upstream 9.57
		scaffold156_1203677	scaffold156	1203677	2.29E-11	Glutathione S-transferase (GST)	downstream 0.52
		scaffold1863_545	scaffold1863	545	8.39E-11	Plant organelle RNA recognition domain (PORR)	upstream 6.46
		scaffold353_773806	scaffold353	773806	7.04E-11	Phosphatidylinositol-4-phosphate 5-Kinase (PIP5K)	downstream 6.62
		scaffold42_494571	scaffold42	494571	1.79E-07	Glycerophosphodiester phosphodiesterase (GP)	interior
		scaffold464_754364	scaffold464	754364	7.77E-07	xyloglucan endotransglucosylase/hydrolase (XTH)	interior
		scaffold635_43971	scaffold635	43971	1.08E-06	Ricinus communis acid phosphatase (RCAP)	interior
		scaffold977_784147	scaffold977	784147	2.69E-10	DNA-damage-repair (DDR)	downstream 1.65
		scaffold359_289139	scaffold359	289139	2.30E-08	Protein of unknown function	upstream 1.25
MLM	2015HRB	scaffold116_30201	scaffold116	30201	3.86E-11	GRAS domain family (GRAS)	upstream 9.57
		scaffold156_1203677	scaffold156	1203677	2.29E-11	Glutathione S-transferase (GST)	downstream 0.52
		scaffold1863_545	scaffold1863	545	8.39E-11	Plant organelle RNA recognition domain (PORR)	upstream 6.46
		scaffold353_773806	scaffold353	773806	7.04E-11	Phosphatidylinositol-4-phosphate 5-Kinase (PIP5K)	downstream 6.62
		scaffold464_754364	scaffold464	754364	7.77E-07	xyloglucan endotransglucosylase/hydrolase (XTH)	interior
		scaffold977_784147	scaffold977	784147	2.69E-10	DNA-damage-repair (DDR)	downstream 1.65
	2016HRB	scaffold977_469888	scaffold977	469888	3.79E-07	Lus10031183.BGIv1.0	upstream 1.2
		scaffold359_282990	scaffold359	282990	7.47E-12	Lus10013155.BGIv1.0	interior
	2016LX	scaffold116_30201	scaffold116	30201	3.86E-11	GRAS domain family (GRAS)	upstream 9.57
		scaffold156_1203677	scaffold156	1203677	2.29E-11	Glutathione S-transferase (GST)	downstream 0.52
		scaffold1863_545	scaffold1863	545	8.39E-11	Plant organelle RNA recognition domain (PORR)	upstream 6.46
		scaffold353_773806	scaffold353	773806	7.04E-11	Phosphatidylinositol-4-phosphate 5-Kinase (PIP5K)	downstream 6.62
		scaffold464_754364	scaffold464	754364	7.77E-07	xyloglucan endotransglucosylase/hydrolase (XTH)	interior
		scaffold977_784147	scaffold977	784147	2.69E-10	DNA-damage-repair (DDR)	downstream 1.65
		scaffold359_289139	scaffold359	289139	2.30E-08	Lus10013156.BGIv1.0	upstream 1.25

#### SNP loci displaying significant correlation with number of fruits

Nine SNP loci exhibited significant associations with the number of fruits (*P* < 1.26E-06). The corresponding Manhattan plots and QQ plots of the two models and three environments are shown in Supplementary Figure 4. The GLM identified three SNP loci in 2015HRB, two SNP loci in 2016HRB, and four SNP loci in 2016LX. Among these, scaffold137_111000 and scaffold225_427119 were identified in both 2015HRB and 2016LX. In addition, scaffold156 and scaffold413 were both identified in 2016HRB and 2016LX, but each had different association loci in the two environments. MLM identified association SNP loci in only two environments, including three SNP loci in 2015HRB and four SNP loci in 2016LX. Among these, scaffold137_111000 and scaffold225_427119 were identified in both 2015HRB and 2016LX. An overview of the results showed that five SNP loci were identified by both models (scaffold137_111000, scaffold225_427119, scaffold687_123666, scaffold156_1203677, and scaffold413_388319). The genes closest to the five SNP loci were *TATP* (Transmembrane amino acid transporter protein), *Contig1437* (*Linum usitatissimum* clone Contig1437 microsatellite sequence), *LU0019C12* (*Linum usitatissimum* clone LU0019C12 mRNA sequence), *FH* (Fumarate hydratase), and *RP* (Ribosomal protein) (Table 5).

**Table 5:** Associated single nucleotide polymorphisms (SNPs) and the nearest genes for the number of fruits trait of flax.

**Model**	**Environment**	**SNP position (bp)**	**scaffold**	**Location**	***P*-value**	**Nearest gene**	**Distance to SNP (kb)**
GLM	2015HRB	scaffold137_111000	scaffold137	111000	6.28E-08	Transmembrane amino acid transporter protein (TATP)	upstream 0.65
		scaffold225_427119	scaffold225	427119	1.91E-07	*Linum usitatissimum* clone Contig1437 microsatellite sequence	downstream 1.53
		scaffold687_121617	scaffold687	121617	7.22E-07	*Linum usitatissimum* clone LU0019C12 mRNA sequence	upstream 0.36
	2016HRB	scaffold156_761294	scaffold156	761294	2.76E-07	Lus10040627.BGIv1.0	downstream 0.54
		scaffold413_1116527	scaffold413	1116527	2.97E-07	Fumarate hydratase (FH)	interior
	2016LX	scaffold137_111000	scaffold137	111000	6.28E-08	Transmembrane amino acid transporter protein (TATP)	upstream 0.65
		scaffold225_427119	scaffold225	427119	1.91E-07	*Linum usitatissimu*m clone Contig1437 microsatellite sequence	downstream 1.53
		scaffold156_1203677	scaffold156	1203677	1.14E-12	Ribosomal protein (RP)	upstream 1.14
		scaffold413_388319	scaffold413	388319	1.03E-06	Chitinase class I (CCI)	upstream 1.02
MLM	2015HRB	scaffold137_111000	scaffold137	111000	6.28E-08	Transmembrane amino acid transporter protein (TATP)	upstream 0.65
		scaffold225_427119	scaffold225	427119	1.91E-07	*Linum usitatissimum* clone Contig1437 microsatellite sequence	downstream 1.53
		scaffold687_123666	scaffold687	123666	3.22E-07	*Linum usitatissimum* clone LU0019C12 mRNA sequence	interior
	2016LX	scaffold137_111000	scaffold137	111000	6.28E-08	Transmembrane amino acid transporter protein (TATP)	upstream 0.65
		scaffold225_427119	scaffold225	427119	1.91E-07	*Linum usitatissimum* clone Contig1437 microsatellite sequence	downstream 1.53
		scaffold156_1203677	scaffold156	1203677	1.14E-12	Lus10040728.BGIv1.0	upstream 1.14
		scaffold413_388319	scaffold413	388319	1.03E-06	Lus10028377.BGIv1.0	upstream 1.02

#### SNP loci showing significant correlation with 1,000-grain weight

Twenty-three SNP loci exhibited significant associations with 1000-grain weight (*P* < 1.26E-06). The corresponding Manhattan plots and QQ plots of the two models and three environments are shown in Supplementary Figure 5. The GLM identified ten SNP loci in 2015HRB, five SNP loci in 2016HRB, and eight SNP loci in 2016LX. Among these, four loci, namely scaffold112_184204, scaffold1143_190268, scaffold1317_154716, and scaffold1519_272169, were repeatedly identified in the three environments; scaffold123_1191347 was repeatedly identified in both 2015HRB and 2016HRB. The MLM identified eight SNP loci in 2015 HRB, seven SNP loci in 2016HRB, and four SNP loci in 2016LX. Among these, four loci, namely scaffold112_184204, scaffold1155_171787, scaffold132_713877, and scaffold1519_272169, were identified in all three environments; eight SNP loci were identified by both models (scaffold112_184204, scaffold123_1191347, scaffold1317_154716, scaffold1519_272169, scaffold1155_171787, scaffold132_713877, scaffold1491_58878, and scaffold15_1207948). The genes closest to the eight SNP loci were *HP* (Hypothetical protein), *NAD-DEF* (NAD dependent epimerase/dehydratase family), *TS* (Terpene synthase), *TPI* (Trypsin and protease inhibitor), *STK* (Serine/threonine protein kinase), *CAP* (CDP-alcohol phosphatidyltransferase), *PHO1* (SPX and EXS domain-containing protein), and *ARP* (Autophagy-related protein) (Table 6).

**Table 6:** Associated single nucleotide polymorphisms (SNPs) and the nearest genes for the 1,000-grain weight trait of flax.

**Model**	**Environment**	**SNP position (bp)**	**scaffold**	**Location**	***P*-value**	**Nearest gene**	**Distance to SNP (kb)**
GLM	2015HRB	scaffold101_354340	scaffold101	354340	3.68E-09	Uncharacterized protein (UP)	interior
		scaffold112_184204	scaffold112	184204	4.55E-09	hypothetical protein (HP)	interior
		scaffold1143_190268	scaffold1143	190268	2.83E-07	serine/threonine-protein kinase (STK)	downstream 0.04
		scaffold1155_171787	scaffold1155	171787	1.23E-08	NAD dependent epimerase/dehydratase family (NAD-DEF)	interior
		scaffold123_1191347	scaffold123	1191347	5.48E-08	Probable terpene synthase (TS)	interior
		scaffold1317_154716	scaffold1317	154716	7.62E-10	Trypsin and protease inhibitor (TPI)	upstream 0.04
		scaffold132_713877	scaffold132	713877	1.52E-11	serine/threonine-protein kinase MPS1-like (STK-MPS1)	interior
		scaffold1491_58878	scaffold1491	58878	3.67E-10	CDP-alcohol phosphatidyltransferase (CAP)	upstream 3.01
		scaffold15_1207948	scaffold15	1207948	3.65E-08	SPX and EXS domain-containing protein (PHO1)	interior
		scaffold1519_272169	scaffold1519	272169	2.52E-10	Autophagy-related protein (ARP)	interior
	2016HRB	scaffold112_184204	scaffold112	184204	5.32E-09	Lus10018116.BGIv1.0	interior
		scaffold1143_190268	scaffold1143	190268	1.61E-07	Serine/threonine protein kinase (STK)	downstream 0.04
		scaffold123_1191347	scaffold123	1191347	3.51E-08	Terpene synthase (TS)	interior
		scaffold1317_154716	scaffold1317	154716	8.00E-09	Trypsin and protease inhibitor (TPI)	upstream 0.04
		scaffold1519_272169	scaffold1519	272169	2.52E-09	Autophagy-related protein (ARP)	interior
	2016LX	scaffold101_354340	scaffold101	354340	3.68E-09	Uncharacterized protein	interior
		scaffold112_184204	scaffold112	184204	4.55E-09	hypothetical protein (HP)	interior
		scaffold1143_190268	scaffold1143	190268	2.83E-07	serine/threonine-protein kinase (STK)	downstream 0.04
		scaffold1155_171787	scaffold1155	171787	1.23E-08	NAD dependent epimerase/dehydratase family (NAD-DEF)	interior
		scaffold1317_154716	scaffold1317	154716	7.62E-10	Trypsin and protease inhibitor (TPI)	upstream 0.04
		scaffold132_713877	scaffold132	713877	1.52E-11	serine/threonine-protein kinase MPS1-like (STK-MPS1)	interior
		scaffold1491_58878	scaffold1491	58878	3.67E-10	CDP-alcohol phosphatidyltransferase (CAP)	upstream 3.01
		scaffold1519_272169	scaffold1519	272169	2.52E-10	Autophagy-related protein (ARP)	interior
MLM	2015HRB	scaffold112_184204	scaffold112	184204	4.55E-09	hypothetical protein (HP)	interior
		scaffold1155_171787	scaffold1155	171787	1.23E-08	NAD dependent epimerase/dehydratase family (NAD-DEF)	interior
		scaffold123_1191347	scaffold123	1191347	5.48E-08	Probable terpene synthase (TS)	interior
		scaffold1317_154716	scaffold1317	154716	7.62E-10	Trypsin and protease inhibitor (TPI)	upstream 0.04
		scaffold132_713877	scaffold132	713877	1.52E-11	serine/threonine-protein kinase MPS1-like (STK-MPS1)	interior
		scaffold1491_58878	scaffold1491	58878	3.67E-10	CDP-alcohol phosphatidyltransferase (CAP)	upstream 3.01
		scaffold15_1207948	scaffold15	1207948	3.65E-08	SPX and EXS domain-containing protein 1 (PHO1)	interior
		scaffold1519_272169	scaffold1519	272169	2.52E-10	Autophagy-related protein (ARP)	interior
	2016HRB	scaffold112_184204	scaffold112	184204	5.32E-09	Lus10018116.BGIv1.0	interior
		scaffold123_1191347	scaffold123	1191347	3.51E-08	Lus10042202.BGIv1.0	interior
		scaffold1317_154716	scaffold1317	154716	8.00E-09	Lus10007888.BGIv1.0	upstream 0.04
		scaffold1519_272169	scaffold1519	272169	2.52E-09	Lus10007527.BGIv1.0	interior
		scaffold132_713877	scaffold132	713877	1.52E-11	serine/threonine-protein kinase MPS1-like(STK-MPS1)	interior
		scaffold1491_58878	scaffold1491	58878	3.67E-10	CDP-alcohol phosphatidyltransferase (CAP)	upstream 3.01
		scaffold15_1207948	scaffold15	1207948	3.65E-08	SPX and EXS domain-containing protein 1 (PHO1)	interior
	2016LX	scaffold112_184204	scaffold112	184204	4.55E-09	hypothetical protein (HP)	interior
		scaffold1155_171787	scaffold1155	171787	1.23E-08	NAD dependent epimerase/dehydratase family (NAD-DEF)	interior
		scaffold132_713877	scaffold132	713877	1.52E-11	serine/threonine-protein kinase MPS1-like (STK-MPS1)	interior
		scaffold1519_272169	scaffold1519	272169	2.52E-10	Autophagy-related protein (ARP)	interior

### Candidate gene prediction

In this study, a total of 42 SNP loci were found to display significant association with five important agronomic traits (*P* < 1.26E-06). The Manhattan plots of the SNP loci (Supplementary Figures 1–5) as well as Tables 2–6 revealed that relatively more SNP loci were found to be linked to number of branches and 1000-grain weight (over twenty for each trait). In comparison, only nine SNP loci showed significant association with plant height or number of fruits, as did only three SNP loci with technical length. Next, candidate genes were screened in the 10 kb zone of each of the 15 SNP loci. The resulting candidate genes were then screened further using co-identification in both the GLM and MLM as well as co-occurrences in at least two of the three environments, whereby 15 final candidate genes were obtained (Table S3).

## Discussion

Genome-wide association study (GWAS) is based on molecular markers, SNPs, that are present throughout a genome and facilitate direct association analysis for complex traits. It is considered an effective method to determine molecular markers that influence crucial traits (Gu et al., 2011; Liu et al., 2013). As a consequence, SLAF-seq-based GWAS has been launched in several crops, such as maize, rice, and soybean. Zhao et al. (2015) used this approach to examine 330 soybean cultivars to identify genes related to resistance against sclerotinia stem rot: the dominant locus *Oswm13-1* was identified and four resistance candidate genes were acquired. Likewise, the method was used to analyze 440 soybean germplasm resources of various origins to identify genes related to resistance against soybean cyst nematode (SCN, *Heterodera glycines* Ichinohe) (Han et al., 2015); the authors identified 19 SNP loci significantly associated with SCN resistance. Su et al. (2016) used SLAF-seq to identify 81,675 SNP loci in cotton, performing GWAS for 355 cotton germplasm resources to identify 11 SNP loci associated with five earliness-related traits. Moreover, Yang et al. (2016) used this approach to analyze 419 core germplasm resources of rice to identify the novel gene *LAC6* as associated with amylose content and show *LOC_Os06g11340* to be a likely candidate gene for *LAC6*. These results indicated that SLAF-seq-based GWAS is a well-developed technology to identify high-quality alleles. In comparison with rice, soybean, maize, and cotton, flax has not received adequate research efforts using GWAS to identify high-quality alleles (no GWAS have yet been reported for flax). In this study, we employed NGS sequencing technology coupled with SLAF-seq to perform GWAS to identify SNP loci associated with important traits and determined their candidate genes.

Population stratification and genetic relationship are two key factors affecting the accuracy of population structure. We used Admixture software to analyze the population structure of flax. The results of population structure analyses showed that flax accessions were clearly divided into three groups—oil using, fiber using and oil-fiber using groups—at *K* = 3. The result indicated a strong divergence between different flax groups. Correspondingly, if the influence of population structure is not considered, then the stratification effect may be misinterpreted as genetic events, leading to pseudo-negativity in the association analysis. Hence, QQ plots of the five important traits under different environmental conditions were generated to validate the accuracy of the population correction. The results of the QQ plots showed that, overall, the observed values matched the expected values except for a few outliers at the ends. In other words, the correction of the population structure produced reliable results; thus, the association analysis did not produce any false associations because of population stratification.

GLM and MLM are the most commonly used algorithmic models in GWAS. The advantage of GLM is that it is more comprehensive and can obtain more SNPs associated with the traits, but its accuracy in identifying SNP loci is worse than MLM (Huang et al., 2010; Yang et al., 2010; Zhang et al., 2010; Liu et al., 2016). MLM can improve the accuracy of the analysis but can also miss some important SNP loci do to the strict screening conditions. Multiple algorithmic models should be used to conduct GWAS data analysis in actual application (Dhanapal and Crisosto, 2013; Hecht et al., 2013; Zhang et al., 2017). However, we found that the observed *p*-value from GLM greatly deviated from the expected *p*-value, while the *p*-value from the MLM model was close to the expected *p*-value (Supplementary Figures 1–5). The results indicated that the false positives were well controlled in the MLM model in our study.

The two association models (i.e., GLM and MLM) and the phenotypic data derived from three environments (i.e., 2015HRB, 2016HRB, and 2016LX) were used to perform GWAS for five important flax traits, generating a total of 107 loci (42 individual SNP loci) that displayed significant association with the five important flax traits (*P* < 1.26E-06). Afterwards, a more stringent screening was performed, in which the 42 SNP loci were subjected to analyses of co-identification by both the GLM and MLM as well as co-occurrence in 2015HRB, 2016HRB, and 2016LX. Ultimately, we identified two SNP loci associated with plant height, six SNP loci associated with the number of branches, five SNP loci associated with the number of fruits, and eight SNP loci associated with the 1,000-grain weight. Given that the aforementioned SNP loci displayed repeated occurrences (in both models and/or in at least two environments), they potentially have pivotal influences on the relevant agronomic flax traits. As such, they can be recruited as candidate genetic markers impacting these five important flax traits. The remaining SNP loci only had single occurrences of association (in only one model and in only one environment); therefore, their reliability must be investigated further.

Next, candidate genes were screened in the 10 kb zone of each of the SNP loci, which generated 15 potential candidate genes. Among these, there were two candidate genes for plant height, *UGT* and *PL*. It was reported that the overexpression of *UGT84B1* and *UGT74E2* in *Arabidopsis thaliana* (*A. thaliana*)causes phenotypes with shorter stature and more shoot branches. *UGT84B1* overexpressors also have wrinkled leaves and reduced root gravitropism (Jin et al., 2013). In addition, *UGT74D1* has been shown to modulate the metabolic pathway of auxin (IAA) in *A thaliana* to influence its development (Tanaka et al., 2014). Transgenic rice lines ectopically over-expressing the *cZOGT1* and *cZOGT2* genes exhibit short shoot phenotypes, delay of leaf senescence, and a decrease in crown root number. These results suggest that cZOGT activity has a physiological impact on growth and development of rice (Kudo et al., 2012). As such, *UGT*, in a similar fashion to its homologs in other plants, is likely involved in developmental regulation in flax, thereby affecting plant height. In addition, studies in rice and *A. thaliana* have shown that *PL* is intricately associated with plant development (Palusa et al., 2007; Leng et al., 2017) and that *PL* promotes plant growth and development via adjusting the cell division rate and cell wall relaxation (Sun and Nocker, 2010; Sun et al., 2010). These reports corroborated our finding that *PL* was a candidate gene for plant height in flax. Association analysis of technical length only identified one gene, *MIF*. Previous studies revealed that *MIF* is related to human disease and immunity (Roberts et al., 2017; Shin et al., 2017); if the gene is overexpressed, it may lead to the expansion and proliferation of cancer cells. However, the gene has not been examined in plants; thus, further studies are needed regarding whether *MIF* indeed affects the technical length of flax. Association analysis of the number of branches identified four candidate genes. Among these, *GRAS* is a transcription factor unique to plants and plays pivotal roles in development and signal transduction. *LS* and *MOC1* are both members of the GRAS protein family. Lack of expression of *LS* prevents the formation of the axillary meristem and in turn decreases the number of axillary buds (Schmitz and Theres, 1999; Greb et al., 2003). Lack of expression of *MOC1* results in the almost complete loss of tillering in rice plants because the gene is responsible for regulating that biological process via promoting the cell cycle (Li et al., 2003; Sun et al., 2010). These findings therefore indicate that *MOC1* is involved in branch formation, directly affects the trait of the number of branches, and is a crucial candidate gene for this trait in flax. In plants, a major function of *GST* is to detoxify exogenous toxins and harmful endogenous metabolites. Specifically, mercapto groups of *GST* can be catalyzed to bind to a variety of endogenous electrophilic compounds and lipophilic substrates (Dixon et al., 2002; Moons, 2003). However, the involvement of GST in the branching or development of plants has not been reported; thus, further studies are needed to verify its association with the number of branches in flax. In *A. thaliana, XTH9* is expressed in the shoot apical meristem of flower buds and flower stalks and is related to the elongation of these tissues; its loss of expression results in a phenotype of short internodal cell length (Hyodo et al., 2003). Moreover, the overexpression of its *Brassica campestris* homolog, *BcXTH1*, in *A. thaliana* leads to the elongation of flower stalks and an increase in plant height (Shin et al., 2006). Hence, the findings in *A. thaliana* studies suggest that *XTH* possibly plays an important role in dictating the number of branches in flax and corroborate our results that *XTH* is a candidate gene for this trait. Of the three genes showing association with the number of fruits, *TATP* has not been reported in plants. In addition, *Contig1437* and *LU0019C12* were both cloned from flax, but there are no studies examining their functions. Therefore, they remain to be validated by functional studies. Of the five candidate genes possibly associated with 1000-grain weight, SPX proteins contain a C-terminal EXS domain, which is a part of the *PHO1* family. In *A. thaliana* and rice, PHO1 participates in transfer and signal transduction of phosphate from roots to the aboveground parts (Hamburger et al., 2002; Svistoonoff et al., 2007; Secco et al., 2010). Because the uptake of phosphorus can clearly improve seed yield in crops, these previous studies on *PHO1* are consistent with our findings that it is an important candidate gene for the 1,000-grain weight. However, *HP, TS, CAP*, and *STK* have not been previously associated with seed yield.

## Conclusion

In this study, we employed SLAF-seq to perform GWAS for five important agronomic traits in 224 germplasm resources of flax. Using two models (i.e., GLM and MLM) for flax grown in three environments (i.e., 2015HRB, 2016HRB, and 2016LX), we identified a total of 42 SNP loci displaying a significant association (*P* < 1.26E-06), including 15 SNP loci having co-identification either by both models or by co-occurrence in two or more environments. Next, candidate genes were screened in the 10 kb zone of each of the 15 SNP loci to identify 15 candidate genes possibly related to the five important agronomic traits. Our subsequent analyses determined that *UGT* and *PL* are candidate genes for plant height, *GRAS* and *XTH* are candidate genes for the number of branches, *Contig1437* and *LU0019C12* are candidate genes for the number of fruits, and *PHO1* is a candidate gene for 1,000-grain weight. These SNP loci and candidate genes may serve as a biological basis for improving these important traits of flax.

## Author contributions

ZD, ZY, LZ, and QT carried out most of the experimental work, and this study was conceived by JSu. Collections of flax germplasm resources were performed by DZ and XY. DX and JSun designed the research and wrote the manuscript. All authors read and approved the final manuscript.

### Conflict of interest statement

The authors declare that the research was conducted in the absence of any commercial or financial relationships that could be construed as a potential conflict of interest.
