# Supplementary material for: Genome-Wide Association Study Identifying Candidate Genes Influencing Important Agronomic Traits of Flax (Linum usitatissimum L.) Using SLAF-seq
Source: Front Plant Sci. 2018 Jan 9;8:2232. doi: 10.3389/fpls.2017.02232 (PMC5767239; doi:10.3389/fpls.2017.02232)
Supplement: Supplementary file 2 [file Table2.DOCX]

**Table S2. Statistic results of SLAF tags and polymorphic markers**

| SLAF number | No. of Polymorphic SLAF | Average depth | No. of Polymorphic SNP |
| --- | --- | --- | --- |
| 346,639 | 146,959 | 7.2 | 584,987 |
