# Supplementary material for: Genome-Wide Association Study Identifying Candidate Genes Influencing Important Agronomic Traits of Flax (Linum usitatissimum L.) Using SLAF-seq
Source: Front Plant Sci. 2018 Jan 9;8:2232. doi: 10.3389/fpls.2017.02232 (PMC5767239; doi:10.3389/fpls.2017.02232)
Supplement: Supplementary file 3 [file Table3.DOC]

Table S3 List of the candidate genes

| **Trait** | **Scaffold** | **Physical position** | **Distance to SNP(kb)** | **Predicted gene** |
| --- | --- | --- | --- | --- |
| Plant height | scaffold344 | 309662 | downstream4.558 | UDP-glycosyltransferase (UGT) |
|  | scaffold51 | 1349321 | downstream8.566 | Pectate lyase(PL) |
| Technical length | scaffold273 | 68457 | interior | Macrophage migration inhibitory factor(MIF) |
| Number of branches | scaffold116 | 30201 | upstream9.57 | GRAS domain family(GRAS) |
|  | scaffold156 | 1203677 | downstream0.52 | Glutathione S-transferase(GST) |
|  | scaffold353 | 773806 | downstream6.62 | Phosphatidylinositol-4-phosphate 5-Kinase(PIP5K) |
|  | scaffold464 | 754364 | interior | xyloglucan endotransglucosylase/hydrolase(XTH) |
| Number of fruits | scaffold137 | 111000 | upstream0.65 | Transmembrane amino acid transporter protein(TATP) |
|  | scaffold225 | 427119 | downstream1.53 | Linum usitatissimum clone Contig1437 microsatellite sequence |
|  | scaffold687 | 121617 | upstream0.36 | Linum usitatissimum clone LU0019C12 mRNA sequence |
| 1000-grain weight | scaffold112 | 184204 | interior | hypothetical protein(HP) |
|  | scaffold123 | 1191347 | interior | Terpene synthase (TS) |
|  | scaffold1143 | 190268 | downstream0.04 | Serine/threonine protein kinase(STK) |
|  | scaffold1491 | 58878 | upstream3.01 | CDP-alcohol phosphatidyltransferase(CAP) |
|  | scaffold15 | 1207948 | interior | SPX and EXS domain-containing protein (PHO1) |
