# Supplementary material for: Genome-Wide Association Study Identifying Candidate Genes Influencing Important Agronomic Traits of Flax (Linum usitatissimum L.) Using SLAF-seq
Source: Front Plant Sci. 2018 Jan 9;8:2232. doi: 10.3389/fpls.2017.02232 (PMC5767239; doi:10.3389/fpls.2017.02232)
Supplement: Supplementary file 4 [file Image1.PDF]

Supplementary Material 1

Genome-wide association study identifying candidate genes influencing important agronomic traits of flax (*Linum usitatissimum* L.) using SLAF-seq

Dongwei Xie[1,2+], Zhigang Dai[1+], Zemao Yang[1], Jian Sun[3], Debao Zhao[2], Xue Yang[2], Liguo Zhang[2], Qing Tang[1], Jianguang Su[1*]

*Correspondence:
Jianguang Su
su_changsha@163.com

2015HRB-GLM

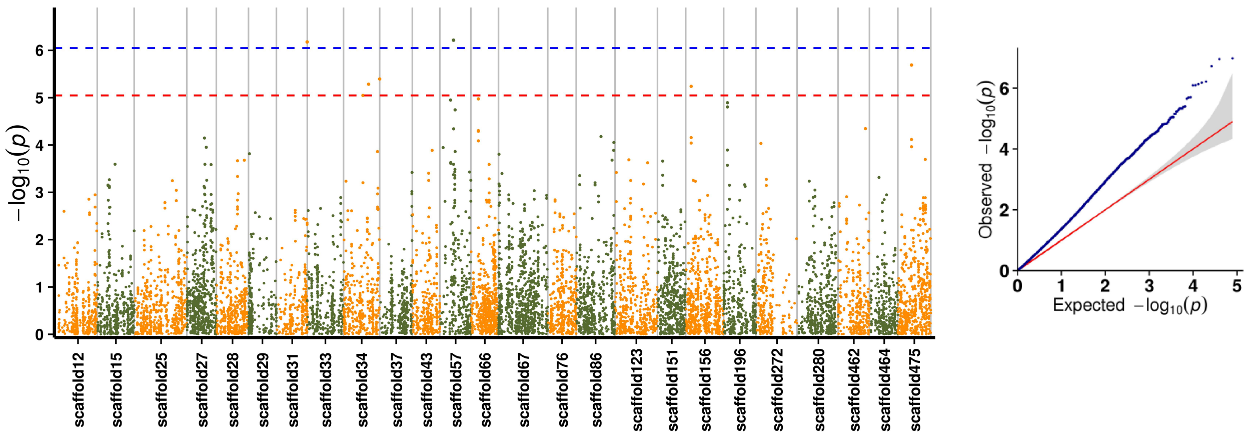

2015HRB-MLM

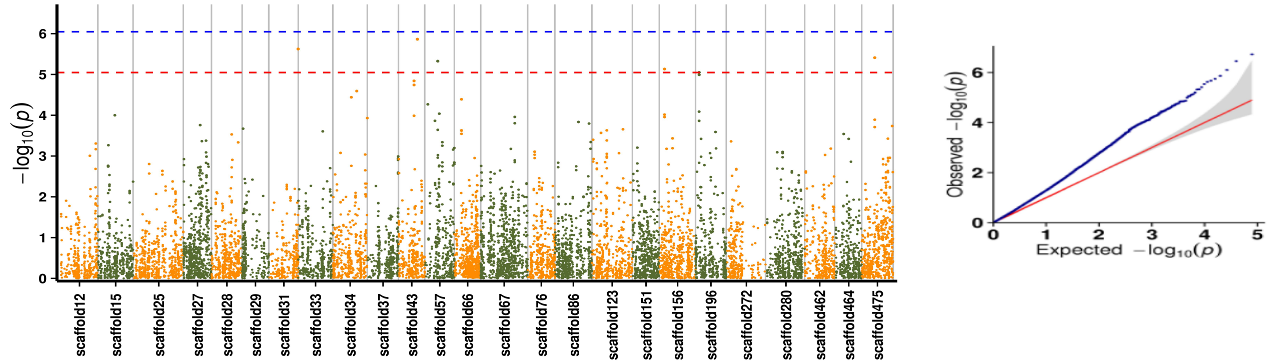

2016HRB-GLM

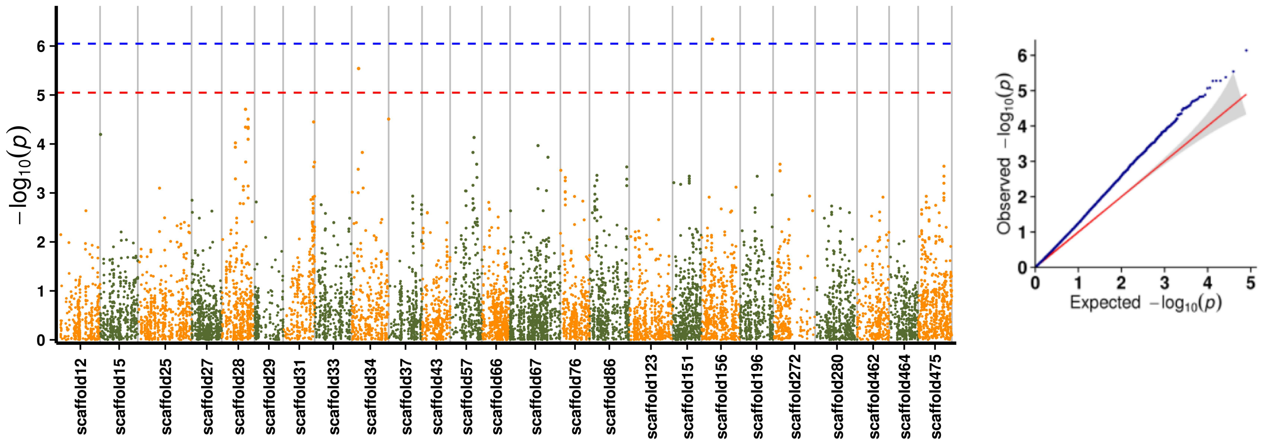

2016HRB-MLM

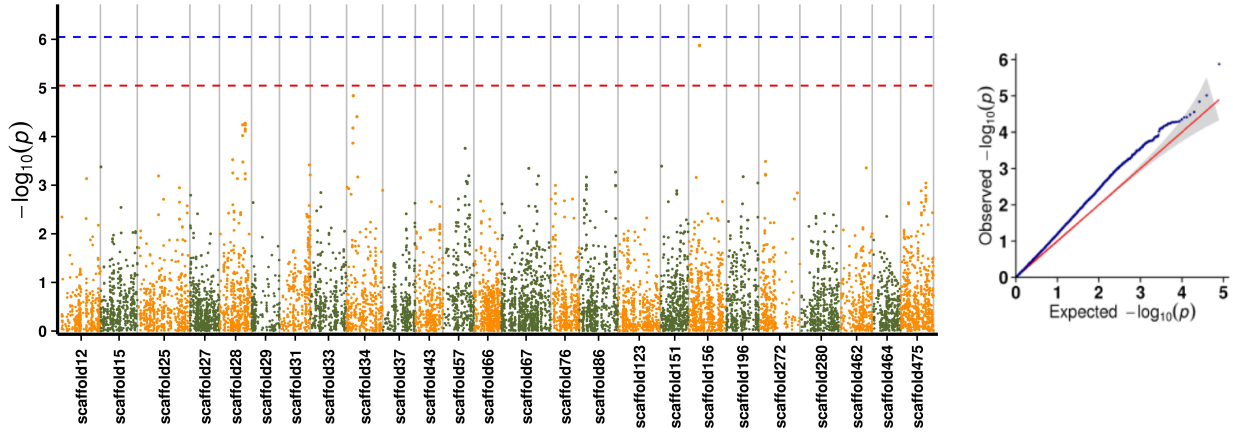

2016LX-GLM

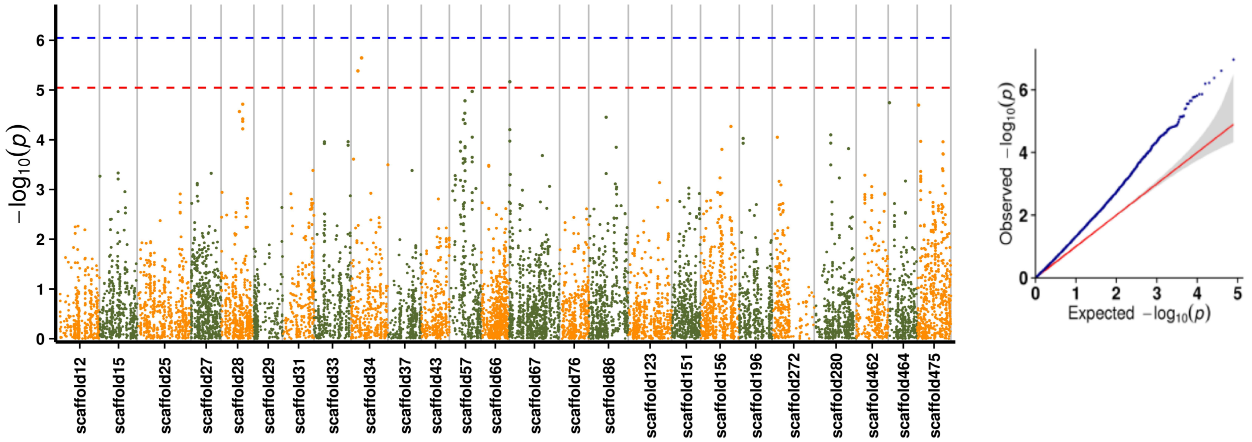

2016LX-MLM

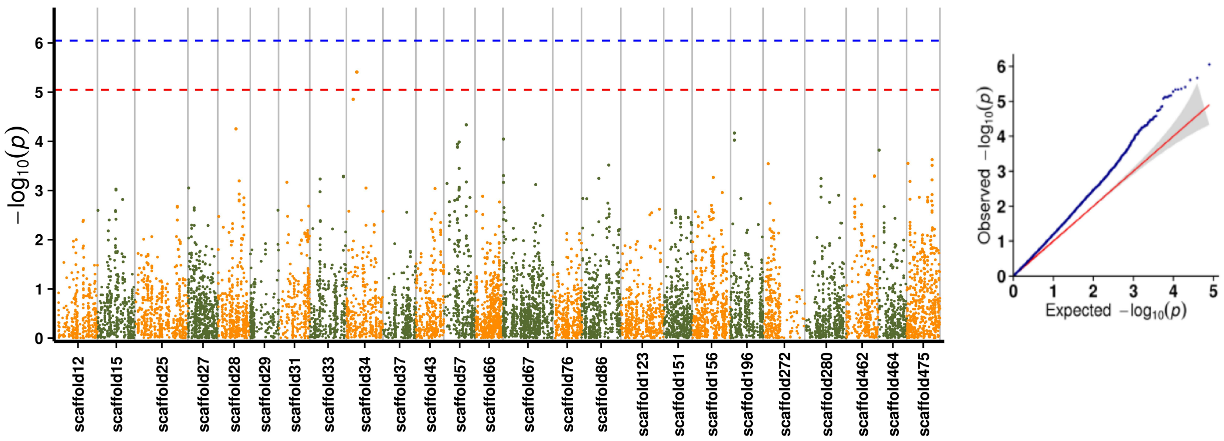

Supplementary Figure 1. Genome-wide association study (GWAS) of plant height in flax. Manhattan plots with the matching QQ plots are shown in the same figure for each of two models (GLM and LMLM) under three environments (2015HRB, 2016HRB and 2016LX). The associated single nucleotide porlymorphisms (SNPs) appear above the line. The Bonferroni multiple test threshold is shown as a imaginary red line ($P \leq 1.268*10^{-5}$) and blue line ($P \leq 1.268*10^{-6}$).
